# Post-tuberculosis lung disease: Exposing the elephant in the room

**DOI:** 10.7196/AJTCCM.2021.v27i2.150

**Published:** 2021-06-23

**Authors:** J A Shaw, B W Allwood

**Affiliations:** 1 DST-NRF Centre of Excellence for Biomedical Tuberculosis Research, South African Medical Research Council Centre for Tuberculosis Research, Division of Molecular Biology and Human Genetics, Faculty of Medicine and Health Sciences, Stellenbosch University, Cape Town, South Africa; 2 Division of Pulmonology, Department of Medicine, Faculty of Medicine and Health Sciences, Stellenbosch University, Cape Town, South Africa

## Editorial

For decades, even centuries, medical practitioners have been aware of the possibility that respiratory symptoms may persist beyond tuberculosis (TB) ‘cure’. In South Africa (SA), we are so familiar with this entity that we recognise the patterns of post-TB lung disease (PTLD) on chest radiography with ease, even when a patient does not report having had TB. Frequently, we have created our own colloquial terminology to deal with the condition, using it freely in patient notes, despite lack of formal representation of these terms in the literature or in our medical textbooks. Respiratory physicians from another sub-Saharan African country reported that within the first 6 months of opening their specialist referral clinic, PTLD was the most common reason for referral.^[1]^ We have all treated these patients in our primary healthcare clinics, and specialist referral centres without even a consistent terminology or definition of disease, let alone the guidance of well-conducted prospective studies or an understanding of the pathophysiology. This is now changing.

TB is now a well-recognised risk factor for chronic respiratory disease. Adults over the age of 40 years who have had TB are three times more likely to have chronic obstructive pulmonary disease (COPD) than those who have not had TB, independent of smoking status.^[2]^ The World Health Organization (WHO) estimates that >60 million lives have been saved through TB treatment programmes since 2000.^[7]^ Perhaps more meaningfully, a recently published modelling study which made use of WHO case notification data estimated that of the 363 million people diagnosed with TB between 1980 and 2019, 155 million people are still alive in 2020, and 18% of them will have been diagnosed and treated within the last five years.^[3]^ Recently, at the 1st International Post-Tuberculosis Symposium, the term ‘post-TB lung disease (PTLD)’ was adopted to unify the myriad of terms used in the international literature to describe the pulmonary impairments after tuberculosis. Similarly, using the Delphi process, the following definition for PTLD was adopted: ‘Evidence of chronic respiratory abnormality, with or without symptoms, attributable at least in part to previous tuberculosis’.^[4]^ This broad definition was proposed as a concession towards inclusivity, while balancing the many clinical complexities of PTLD with limited available longitudinal research, and is likely to be refined over time.

Apart from symptoms and spirometry abnormalities, PTLD can be considered in terms of: an airway component, namely bronchiectasis (large airways) and tuberculosis-associated obstructive lung disease (TOPD or small airway disease); a parenchymal component namely cavitation, destruction, fibrosis and aspergillus-related disease; as well as pulmonary vascular and chronic pleural components.^[4]^ Some or all of these may be seen in any one individual, yet the long-term effects of these well-recognised patterns are not known. Most importantly, despite successful treatment, merely having had TB increases all-cause mortality, reduces life expectancy and predisposes to further episodes of recurrent TB disease.^[5–7]^ Disability-adjusted life-years (DALYs) is a framework which measures the years of life lost to premature death as well as years lived with disability. To date, the WHO estimates of DALYs associated with TB have not included the post-TB period. A recent study conservatively estimated an increase of 6.1 million DALYs through the elevated prevalence of COPD, which was attributable to PTLD in India alone, representing a 54% increase over estimates that consider end-of-treatment to be a return to health.^[8]^ In an SA study,^[9]^ patients admitted to hospital with a diagnosis of chronic lung disease who reported a previous episode of TB were significantly more likely to have recurrent hospital admissions, and to have a longer stay in hospital than their counterparts who had no known TB episode. Despite these data, outcomes beyond the end-of-treatment are still not considered in the rubric against which TB prevention programmes and policies are judged.

In this issue of the *AJTCCM*, a study by Ozoh *et al*.^[10]^ characterised in some detail a cohort of 59 patients who received treatment for PTLD in respiratory specialist clinics in Lagos, Nigeria. The patients described were non-smokers, or ex-smokers with a less than 10 pack a year history, and only 8.5% (n=5) were living with HIV, minimising the impact of these two important confounders to a large degree. They were a young group with only a quarter of patients aged over 60 years old. Females were slightly over-represented. Importantly, the majority of these patients had suffered recurrent episodes of TB. Almost two-thirds of participants (62.4%; n=38) were treated for TB more than once, and 23.7% (n=14) had received treatment three or more times. Although this variable has not been consistently associated with post-TB outcomes, logic suggests that recurrent episodes should compound the lung damage.^[11,12]^ Indeed, what is described here is a group with physiologically severe, symptomatic chronic lung disease. The symptom burden was high, with over 90% of patients reporting cough and sputum production, half of whom had haemoptysis. More than 50% of patients had a modified Medical Research Council (mMRC) grading of 2 or higher. Three-quarters of patients (75.5%; n=40) who had spirometry had severe or very severe impairment on post-bronchodilator forced expiratory volume in 1 second. The St George’s respiratory questionnaire (SGRQ) mean score was 36, with participants scoring highest in the symptoms category. Imaging revealed that 61% of participants had bronchiectasis, 28.8% had destroyed lungs, 18.6% had aspergillomata and 8.4% had atelectasis. Although the criteria for the diagnosis are not stated, 5% of participants had a diagnosis of cor pulmonale. Interestingly, there were some significant associations between the SGRQ and spirometry parameters on bivariate analysis, unlike in other prospective studies.^[11]^

Ozoh et al.^[10]^ presents in this issue a small sample of highly selected patients that were recruited on the basis of self-presentation and are likely not to represent the global population of individuals post-TB. Certainly in other studies performed in the sub-Saharan community, the estimates of symptoms and burden of disease was lower than these estimates. A study conducted by Meghji *et al*.^[13]^ followed first-episode TB treatment survivors in Blantyre, Malawi for one year after the end-of-treatment. They described a young cohort (median age 35 years) of 405 patients with a high proportion of HIV infection, socioeconomic deprivation, and biomass fuel exposure, which had a high burden of PTLD. The proportion of participants who suffered from severe disease was small in this population. At one year after treatment, 12% of patients described respiratory symptoms which interfered with work, and 4.3% reported symptoms which prevented any activity. Four patients had hypoxaemia at rest, and three had pedal oedema.^[13]^ A cross sectional study in suburban Cape Town, SA interviewed 51 patients who had completed TB treatment a median of 2.6 years before, 35% of whom reported two or more episodes of TB.^[14]^ Almost half were living with HIV. More than half of the patients reported dyspnoea of mMRC grade 2 or more, and 29% were supported by a disability grant. Almost a third of the patients (29%) had experienced a severe respiratory illness in the previous year. Another cross-sectional survey in a different region of Cape Town, SA, examined young adults who completed TB treatment within the previous five years, and 38% of these participants had more than one episode of TB. A quarter of the patients (25%) reported mMRC grade 3 or 4 dyspnoea. The impact of recurrent episodes of TB on the presentation and severity of PTLD in these studies is not understood. Similarly, it is still unclear how measured variables, such as spirometry and symptoms relate to both functional status and long-term outcomes such as exacerbation frequency and mortality. In the paper in this issue of the journal, Ozoh *et al*.^[10]^ did not report on the patient’s 6-minute walk distance. But even where this has been done, frequently the associations with symptoms and spirometry can be disappointing.^[11]^ Nonetheless, this study by Ozoh *et al*. ^[10]^ provides us with a snapshot of the more severe end of the spectrum of PTLD – those patients that we as pulmonologists are likely to encounter. Taken together with existing community data, this shows us that the overall burden of PTLD is high, and a small but significant proportion of these patients will have severe life- and activity-limiting disease. For too long, we as pulmonologists working in high-burden TB settings, have managed to ignore the elephant in the room, which potentially may be one of the most important causes of chronic lung disease worldwide.^[15]^ Robust global and regional estimates of PTLD are sorely needed to drive advocacy and policy. The call is out for respiratory physicians to give this disease entity the attention it deserves, fight against the nihilism, and find a way to prevent and treat PTLD.
